# A randomised controlled trial to compare opt-in and opt-out parental consent for childhood vaccine safety surveillance using data linkage: study protocol

**DOI:** 10.1186/1745-6215-12-1

**Published:** 2011-01-04

**Authors:** Jesia G Berry, Philip Ryan, Annette J Braunack-Mayer, Katherine M Duszynski, Vicki Xafis, Michael S Gold

**Affiliations:** 1 Discipline of Public Health, The University of Adelaide, Adelaide, South Australia; 2 Discipline of Paediatrics, The University of Adelaide, Adelaide, South Australia; 3 School of Population Health and Clinical Practice, The University of Adelaide, Adelaide, South Australia

## Abstract

**Background:**

The Vaccine Assessment using Linked Data (VALiD) trial compared opt-in and opt-out parental consent for a population-based childhood vaccine safety surveillance program using data linkage. A subsequent telephone interview of all households enrolled in the trial elicited parental intent regarding the return or non-return of reply forms for opt-in and opt-out consent. This paper describes the rationale for the trial and provides an overview of the design and methods.

**Methods/Design:**

Single-centre, single-blind, randomised controlled trial (RCT) stratified by firstborn status. Mothers who gave birth at one tertiary South Australian hospital were randomised at six weeks post-partum to receive an opt-in or opt-out reply form, along with information explaining data linkage. The primary outcome at 10 weeks post-partum was parental participation in each arm, as indicated by the respective return or non-return of a reply form (or via telephone or email response). A subsequent telephone interview at 10 weeks post-partum elicited parental intent regarding the return or non-return of the reply form, and attitudes and knowledge about data linkage, vaccine safety, consent preferences and vaccination practices. Enrolment began in July 2009 and 1,129 households were recruited in a three-month period. Analysis has not yet been undertaken. The participation rate and selection bias for each method of consent will be compared when the data are analysed.

**Discussion:**

The VALiD RCT represents the first trial of opt-in versus opt-out consent for a data linkage study that assesses consent preferences and intent compared with actual opting in or opting out behaviour, and socioeconomic factors. The limitations to generalisability are discussed.

**Trial registration:**

Australian New Zealand Clinical Trials Registry ACTRN12610000332022

## Background

Options for consent that are available for health and medical research involving human subjects are: no consent, using either identifiable or non-identifiable data; opt-in consent, where each person is informed about the research and their consent is sought; and opt-out consent, where each person is informed about the research and included unless they indicate an unwillingness to participate [1]. A request for consent may be either project-specific, extended (for future research projects) or broad authorisation for research use [2]. Under the opt-in approach, the subject's failure to act leads to non-inclusion; but 'non-participation' may not stem from a meaningful decision and may reflect a lack of contemplation or intention [3]. Under the opt-out approach, inclusion in research may largely depend on individuals' inertia; therefore, the true proportion of people who do not wish to participate may be understated [4].

In Australia, the *National Statement on Ethical Conduct in Human Research *[2] guides Human Research Ethics Committees (HRECs) to require opt-in consent under most circumstances. However, health information can be used in the conduct of specific activities (including research of various types) without a subject's permission 'provided an assessment is made by an HREC that the research and other activities are, on balance, substantially in the public interest' [5]. Data linkage is one such specific activity [2], defined as 'the bringing together, from two or more different sources, data that relate to the same individual, family, place or event' [6].

The development in recent decades of integrated electronic administrative healthcare databases has enabled sophisticated and powerful population-level data linkage studies on the factors influencing health and wellbeing, and health services evaluation [7-10]. Privacy advocates have perceived these developments as a potential threat to privacy, sparking an increase in the rigour and complexity of the privacy framework in Australia and associated regimens of HREC submissions [5,8,11-14]. Threats to privacy are minimised when data linkage adheres to the best practice protocol [15], whereby strict separation of individual demographic identifiers from clinical health information is maintained during and after the linkage process, ensuring researchers never receive personal identifiers and data custodians never exchange identifiable health data [6,15]. Despite the availability of privacy-conserving linkage protocols, some data custodians still require each individual's opt-in consent for release of data [11,16], with severe adverse consequences for the quality and validity of research. Holman *et al*. suggest that when a system of consent leads to participation rates of less than 90%, the information available for the research becomes biased [11].

Several cross-sectional surveys and focus groups conducted internationally [17-22] and in Australia [5,23] have shown that the public has a strong preference to be asked for consent for health and medical research, including for data linkage studies [5,20,22]. There are some notable exceptions: in two cross-sectional surveys conducted in the United Kingdom [24] and Australia [25], the majority of the public did not consider the inclusion of identifiable health data in a cancer registry and birth defects registry without consent to be an invasion of privacy and expressed support for statutory case registration. In terms of consent to data linkage, there are no studies that have compared the numbers and characteristics of participants enlisted under opt-in and opt-out conditions using a well-designed Randomised Controlled Trial (RCT). While there are RCTs that relate to other aspects of medical research [26-30], the extent of participation has varied widely, ranging from 48%-85% in the opt-in arm and 59%-100% in the opt-out arm, and all but one [28] had a small sample size or flawed methodology [26,27,29,30] (Table 1). Only two RCTs [26,30] are relevant to data linkage in that participation required no effort on the part of the subject in terms of clinic attendance or involvement in disease screening, and there were no follow-up reminders, which are not economically or logistically feasible for large population-level studies [11].

**Table 1:** RCTs of opt-in and opt-out consent

Study population and purpose	**Parents in a health district of the UK were asked for consent for inclusion of low birth-weight infants on a register for the purpose of monitoring disability in children **[26]	**Mothers in the US were asked for consent for inclusion of infants at high risk to participate in a clinical trial of primary follow-up care **[27]	**Angina patients in two general practices in the UK were asked for consent to be involved in clinical research **[28]	**Patients aged 50-74 years in a general practice in Australia were asked to consent to testing decision aids for the screening of colorectal cancer **[29]	**Cancer patients in the Netherlands who had undergone primary surgery were asked for consent for the storage of excised tissue for future research purposes **[30]
Sample size randomised (n)	Opt-in: 39 Opt-out: 30	Opt-in: 32 Opt-out: 25 (3 were excluded as they did not receive the allocated intervention)	Opt-in: 252 Opt-out: 258	Opt-in: 92 Opt-out: 60	Opt-in: 60 'Opt-out plus': 73 Control group (standard opt-out): 131
Mode of invitation	Verbal information, letter and reply slip given by a nurse prior to an infant's discharge from hospital	Verbal information and reply form given by a nurse within 24-48 hours of delivery. The opt-out form was shortened to include only specific disclosures that are appropriate for low risk research	Letter, information leaflet and reply card sent from a doctor	Letter sent from a doctor (plus reply card for the opt-in arm only)	Verbal information, specific information leaflet and reply form given by a doctor/nurse. The control group was only given a routine hospital leaflet and did not receive verbal information
Mode of response	Reply-paid slip	Reply form was collected from the mother	Reply card or telephone	Telephone or email (or reply-paid card for the opt-in arm)	Reply-paid form. The control group leaflet instructed patients to opt out by informing their doctor
Reminder letter	No	No	After two weeks for the opt-in arm only	No	No
Time to respond	Not stated	Prior to discharge from hospital. Once a mother reached a decision, an interview occurred within the next 24 hours (usually 2 hours)	Opt-in: Not stated Opt-out: patients could opt out verbally when telephoned after two weeks	Not stated	One month
Participation rate	Opt-in: 79% Opt-out: 97%	Opt-in: 75% Opt-out: 91%	Opt-in: 48% Opt-out: 59%	Opt-in: 51% Opt-out: 90%	Opt-in: 85% 'Opt-out plus': 97% Standard opt-out: 100%
Recruitment rate	Not applicable	Face-to-face interview Opt-in: 81% Opt-out: 82%	Clinic attendance Opt-in: 38% Opt-out: 50%	Telephone survey Opt-in: 47% Opt-out: 67%	Postal and telephone survey Opt-in: 93% and 52% 'Opt-out plus': 93% and 51% Standard opt-out: 88% and 47%
Evidence of selection bias	Not stated	Modest differences were found. Subjects recruited in the opt-in arm were older, more likely to be married and undergo a vaginal delivery than subjects in the opt-out arm	Subjects recruited in the opt-in arm were healthier and had less risk factors for coronary disease than subjects in the opt-out arm	Subjects recruited in the opt-in arm were more likely to prefer an active role in decision making than subjects in the opt-out arm	Subjects recruited in the opt-in arm were similar in age, sex, education and type of cancer to the 'opt-out plus' arm. The control group was similar, except that women were over-represented
Design flaws	Small sample size, non-random allocation and no mention of whether blinding was used	Small sample size and the collection of reply forms is resource-intensive and impracticable on a large scale	None evident	Small sample size and non-parallel design	Small sample size and no mention of whether blinding was used

There has been relatively little research on non-participants in RCTs because of problems in obtaining ethical approval [31,32]. Only two RCTs have elicited the intent behind the return or non-return of forms for subjects in the opt-in and opt-out trial arms by means of a face-to-face interview [27], or postal and telephone survey [30]. We designed a large RCT of opt-in and opt-out consent for a proposed data linkage study into adverse events following immunisation. All eligible subjects were included in the RCT without their prior consent being sought, which necessitated a consent waiver from the approving HREC. Our justification for not obtaining consent was that if prior consent were sought it would lead to a selection bias in the study sample. In order to study reasons for participation and non-participation, we followed up the trial with a telephone interview aimed at all randomised subjects, whether or not they had indicated consent to the data linkage study.

### Purpose

#### Primary objective and hypotheses

To determine which method of obtaining parental consent (opt-in or opt-out) provided the highest participation rate for a population-based childhood vaccine safety surveillance program using data linkage.

The following Null hypotheses will be tested:

(1) There is no difference in the participation rate for the opt-in and opt-out method, that is, the proportion of parents who opt in by return of a reply form (or telephoning or email) and the proportion who do not opt out.

(2) Neither the opt-in nor opt-out method of consent will result in parental participation greater than 90%.

#### Secondary objective and hypotheses

To examine consent preferences, and attitudes and knowledge about vaccine safety, data linkage and vaccination practices by means of a structured telephone interview of all randomised subjects.

The following Null hypotheses will be tested:

(1) There are no differences in the motivations and barriers given for the return/non-return of the reply form by subjects who consented, or did not consent, in the opt-in arm compared with subjects in the opt-out arm.

(2) There are no differences in consent preferences, and attitudes and knowledge about vaccine safety, data linkage, vaccination practices and socio-demographics of subjects who consented, or did not consent, in the opt-in arm compared with subjects in the opt-out arm.

## Methods and Design

### Study design and flow

This was a single-centre, stratified (firstborn versus subsequent births), single-blind, parallel-group RCT conducted in the Women's and Children's Hospital (WCH), a tertiary referral centre in metropolitan Adelaide, the capital city of South Australia (SA) with a population of about 1.19 million in 2009 [33]. Approximately 25% of all South Australian babies are delivered at the hospital [34]. The study population consisted of parent(s) of every consecutive child born in a three-month period: from July 27, 2009, to October 25, 2009, inclusive. Data listings of eligible live births were provided by the SA Department of Health (SA Health) utilising the electronic patient management system (*HOMER™*). The RCT received ethical approval from the Children, Youth and Women's Health Service (CYWHS) HREC (Reference: REC2087/7/11) who granted a waiver of the usual requirement of individual, fully informed consent to participate in an RCT and allowed the limited disclosure to subjects of the true purpose of the trial.

### Inclusion and exclusion criteria

**Figure 1:**
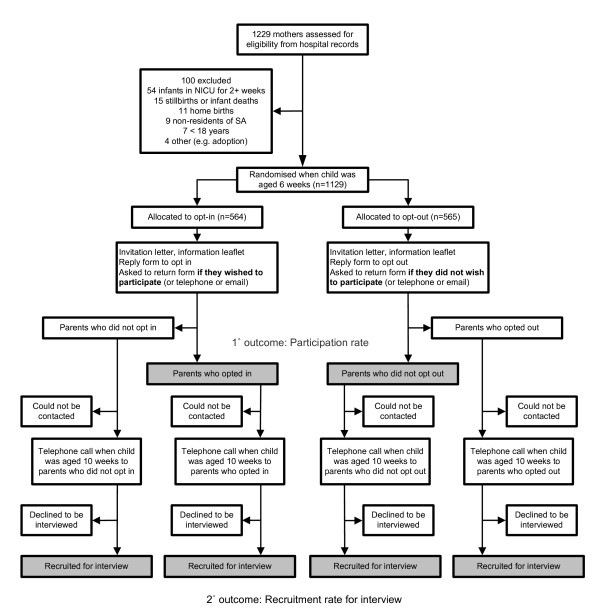
**Flow diagram of opt-in compared with opt-out trial**.

Selection of subjects was based on the hospital records of mothers who met the study eligibility requirements (Table 2). Further exclusions were made on a case-by-case basis if an audit of the medical record revealed that the mother was incarcerated, mentally incapacitated, or the baby had been adopted or placed into foster care. Since infant (and maternal) deaths following a mother's discharge are not routinely captured in the hospital's patient management system, the South Australian Births, Deaths and Marriages Registration Office was engaged to conduct weekly searches to identify any deaths which might have occurred prior to randomisation and, where identified, the mother was excluded from the trial. Weekly searches for deaths continued until parents exited the interview. The flow of subjects in this study is shown in Figure 1.

**Table 2:** Eligibility criteria and rationale

Criterion	Rationale
*Inclusion criteria*	
Mothers who had a live and surviving birth at the WCH.	A birth must be viable and surviving to enable data linkage of immunisation encounters at two months and hospital admissions after birth.
Mother's age was equal or above 18 years.	This is the age accepted by HRECs where informed consent can be given by an individual.
Mother was a resident of SA.	The data linkage will involve only South Australian children whose immunisation encounters will be linked with admissions to a South Australian hospital. Cross-jurisdictional migration will be unaccounted for i.e., if a family moves interstate after the birth or an infant is admitted to an interstate hospital.
*Exclusion criteria*	
Maternal death, stillbirth or neonatal death. In the instance of twins or triplets, if one died, the mother was excluded.	To avoid causing distress to a bereaved family.
Infant stays in the NICU of 2 weeks or longer.	To avoid causing distress to a family dealing with issues of infant illness and prematurity.
Home births and births that occurred at other hospitals and were subsequently managed at the WCH.	To ensure each mother had received the same type of care prior to discharge and data were available in the hospital patient management system for all variables of interest.

### Sample size

The primary outcome of interest was a comparison of the parental participation rate in each arm. To detect an effect size difference of 10% (assuming 80% in the opt-out arm and 70% in the opt-in arm) using a two-tailed test at the 5% level with power of 80% we required 313 subjects in each arm (total 626 subjects). A further 10% inflation allowed for the stratified design of randomisation for the pre-specified confounder of firstborn status. Thus, the sample size for the primary outcome required 344 subjects in each arm: a total of 688 subjects. However, important secondary outcomes of interest related to the recruitment of parents for the subsequent telephone interview. The sample size required for the secondary outcome was 544 subjects in each arm: a total of 1,088 subjects.

### Randomisation and blinding

The unit of randomisation was the mother who was randomly allocated, by date order of confinement, to the opt-in and opt-out arm in the ratio 1:1. The randomisation schedule was stratified by firstborn status (first live and surviving birth versus subsequent births). It used randomly permuted blocks of sizes 2, 4, 6 and 8 and was created using the program ralloc [35] in Stata statistical software [36]. We stratified on firstborn status because we judged that a parent's likelihood of participating in a data linkage study of childhood vaccine safety, and their attitudes towards vaccination and vaccination practices, could be influenced by previous experience of infant immunisation, especially if an adverse event occurred following immunisation.

The trial was single-blinded: parents were unaware that two types of consent were being compared, but were aware of the data linkage study. Blinding was not appropriate for the two researchers [JGB, JW] who conducted interviews since the interview structure required knowledge of whether the parent had, or had not, returned the reply form. For the analysis and reporting, the primary outcome will be assessed by one researcher who will be blind to allocation.

### Intervention and follow-up

All households received a cover letter (addressed to the mother), an information leaflet and a reply-paid form, with different formats according to allocation to the opt-in or the opt-out arm (Figure 1). The study material invited parents to be part of a 'Vaccine Data Linkage Study' in order to investigate data linkage as a new way of checking for rare reactions to vaccines by looking at large numbers of children. Parents were asked for permission to link infants' two-month vaccination records with any hospital visits occurring in the month following vaccinations. The study invitation was mailed after randomisation at six weeks post-partum; its arrival was timed to advise parents of the study one to two weeks prior to the scheduled two-month vaccinations. Parents in the opt-in arm were instructed to return a reply form to signal willingness to participate in data linkage; whereas parents in the opt-out arm were informed they would be included unless they returned a reply form to refuse consent. Telephone or email response was also accepted. No follow-up reminder letters were sent, to make the participation rate - the response to one invitation - relevant to large population data linkage studies. The cut-off time for data to be included in the estimation of the participation rate at 10 weeks post-partum included the first day of the 11th week to allow for internal hospital postal delays, giving all parents four weeks to respond. The telephone interview occurred when infants reached 10 weeks of age, corresponding with one to two weeks after administration of the two-month vaccinations to enable data collection on vaccination practice and experience of adverse events.

### Data management

All data collection and interviews occurred at the study centre. A database was developed to manage the study flow and follow-up of subjects, the mail-out of study invitation material, and transcription of telephone interview responses from paper booklets into electronic format. All data were kept securely on a non-networked computer. File back-ups and associated paperwork were stored in a locked filing cabinet, as required by relevant guidelines for the ethical conduct of research.

### Outcome assessment

The primary outcome at 10 weeks post-partum was the proportion of parental participation in each arm, as indicated by the respective return or non-return of a reply form (or via telephone or email response). Secondary outcome data, including socio-demographic characteristics, were captured from the hospital's patient management system and at the subsequent telephone interview at 10 weeks post-partum. These included: 1) the interviewee's age, gender, marital status, country of birth, main language at home and level of education; 2) the mother's age, marital status, country of birth, Indigenous status and firstborn status of the infant; and 3) the household size, composition, annual income, Socio-Economic Indexes For Areas (SEIFA) Index of Relative Socio-Economic Disadvantage (IRSD) [37] and location (major cities or other). The IRSD and location measures were derived from postcode of usual residence.

The study invitation material and telephone survey were designed and administered according to recommended principles [38,39]. They were initially piloted on a small number of academic staff, and then further modified and refined through piloting on a convenience sample of 20 subjects similar to the study's target group: parents of young children. The pilot groups were re-utilised by the study team for training purposes to develop skills in the delivery of the telephone interview. The survey collected information on the parent's recall of the study and its purpose, reasons for the return or non-return of the reply form, consent preferences, understanding of data linkage and the level of trust in its protection of privacy. The survey also canvassed attitudes towards vaccination in terms of its public health benefit, safety, and effectiveness; vaccination practices; experiences of minor and serious infant illness and the likelihood of being vaccine-related; and socio-demographics measures. Questions relating to consent preferences [20] and perceptions about the safety and effectiveness of vaccines [20,40] were derived from published telephone surveys to assist in comparison with similar studies.

The interview schedule was designed to be pragmatic to optimise response rates. While the researchers endeavoured to interview the parent (either mother or father) who had opted in or opted out as identified by name on the reply form, this was not always possible even with multiple call-backs. In such instances, the other parent, if available, was interviewed as a proxy. For households who neither opted in nor opted out, the interviewers had no knowledge of which parent, if any, had read the study invitation material. The first parent to answer the telephone was invited, as there was no basis for preferentially interviewing one parent over the other.

### Analysis plan

All analyses will be performed on an intention-to-treat basis. The primary outcome, consent to participate, will be compared using a chi-square test, modified appropriately (Mantel-Haenszel method) to account for the permuted block randomisation. The Type I error level is set at 0.05 (two-tailed). There are no pre-specified confounders for the primary analysis. Comparisons of socio-demographic characteristics between those consenting in the two arms will use chi-square tests, t-tests or Wilcoxon rank sum tests appropriate to the scale of measurement. The secondary outcomes for the study arms will be compared using simple tests (as above) and adjusted for socio-demographic characteristics where appropriate using generalised linear models. Missing data are likely and, if missingness is considered to be either at random or completely at random, multiple imputation will be used. A total of 50 imputed datasets will be generated using the package mi in Stata statistical software [36]. Depending on the pattern of missing values, we will use sequential univariate conditional distributions or a multivariate normal method, using socio-demographic and other background variables as predictors. The quality of the imputations will be evaluated by checking how reasonable the imputed data are and testing the fit of the missing-data models.

### Recruitment

Procurement of subject lists was straightforward and timely, with exclusions readily identifiable from existing data fields (Figure 1). Six ineligible mothers were included in the trial as a result of recording errors in the data fields (e.g., mothers whose infants had had an extended Neonatal Intensive Care Unit (NICU) stay or mothers who were non-residents of SA). An additional five mothers included in the trial would have been excluded had it been possible to audit all the medical records prior to randomisation. Examples were mothers who were incarcerated, mentally incapacitated, or whose baby was placed into foster care. If the mother's exigent circumstances were discovered upon audit of the medical record (to follow-up returned mail or non-contacts for the telephone interview), no further contact was attempted.

### Implementation of telephone interviews

Households were initially contacted on the day the infant reached 10 weeks of age. A minimum of three calls was made at varying times of the day (morning, afternoon, evening) before a household was classified as non-contactable. Interview recruitment was high: 1026 parents were interviewed (91%), of whom 925 (82%) completed the interview and 101 (9%) partially completed the interview. A partially completed interview was usually one where the parent answered one question only: the reason why they did or did not return the reply form. There were 57 non-contactable households (5%) despite repeated attempts and a connected telephone number. A further 13 households (1%) were non-contactable due to disconnected or wrong telephone numbers recorded in the hospital system, while 28 (2%) were non-English speaking and 5 (< 1%) refused to be interviewed.

## Discussion

This is the first RCT of opt-in and opt-out parental consent for a population-based childhood vaccine safety surveillance program using data linkage. It featured a parallel design, adequate power for the primary outcome and thorough follow-up of subjects to determine attitudes to consent, data linkage and other important issues.

The comprehensive list of socio-demographic variables included in the hospital's patient management system provided basic information on a range of socio-demographics for all mothers: age, marital status, country of birth, Indigenous status, household location (major cities or other) and IRSD. It will, therefore, be possible to determine the presence of selection bias in the participation rate, irrespective of whether a subject answered the socio-demographic questions in the interview. The previous RCTs of opt-in versus opt-out consent [26-30] did not show such comparisons [26,27], or were restricted by the small number of socio-demographic characteristics for which data were available for all eligible patients: either age and sex alone (which gave no insight) [28,30], or age, sex and the IRSD (which gave limited insight) [29].

The recruitment rate for the interview was high for a number of reasons. Firstly, SA Health's data listings recorded a mother's mobile and landline telephone number and often a spouse or *de facto's *mobile number, enabling parents to be contacted even if they had changed residence. Secondly, the interviewers were persistent in the follow-up of returned mail and non-contacts for the interview, and optimised contact through auditing medical records to find valid residential addresses and telephone numbers. Thirdly, parents usually had a good rapport with the hospital as recent recipients of its health services and were willing to participate in the research for altruistic reasons.

This trial focused on parental attitudes towards using data linkage in one context: childhood vaccine safety surveillance. Although there will be some overlap in motivators and barriers to participation, some important determinants of participation among parents may not be relevant for data linkage studies in other health-related areas. The portfolio of evidence on the public's preferences for consent and attitudes towards the intrinsic value of data linkage, levels of trust in its protection of privacy in different population/patient groups and in different health areas requires expansion.

The cut-off time for data to be included in the estimation of the participation rate was chosen *a priori *to allow parents sufficient time to immunise their infants, and for adverse events to be captured, and to balance the potential for recall bias against potential for late returns. Every parent had four weeks to respond to the study invitation material and did not receive follow-up reminders. Based on findings from previous surveys [38,39], we anticipate that the number of parents who opted in or opted out may be half those attained if follow-up mailings had been implemented. We accepted reply forms that were received within a week of the interview at 10 weeks post-partum, which prompted a small number of crossovers. For example, a parent in the opt-in arm may have answered in the interview that they had been too busy to send back the reply form, but the process of being interviewed reminded them to do so. In this instance, it is unlikely the parent would have returned the reply form of their own volition.

The interviews may have been subject to respondent bias, in that parents may not have honestly reported motivations and barriers to the return or non-return of reply forms in a telephone conversation with a 'stranger' affiliated with the trial. While qualitative methodology may be more successful in revealing true motive, facilitated by developing rapport with interviewers and/or focus group members through in-depth exploration of reasons underlying participation and non-participation, fewer parents could have been studied in the same time.

We did not engage interpreter services for non-English speakers. While the number of parents who had no English comprehension was smaller than anticipated (2%), we encountered parents with varying levels of English proficiency, ranging from the ability to comprehend and answer a small number of questions in the interview (usually only questions related to vaccinations practices and episodes of infant illness) to answering all questions, but with some uncertainty as to their understanding. The interviewers flagged the interviews in which they perceived the parents' English to be limited, and this can be used as a covariate in the analysis, in addition to the socio-demographic variables that provide information on country of birth and main language spoken at home.

Informed consent is generally regarded as an essential component of health research. Low participation rates in health and medical research can lead to selection bias and compromise statistical precision. Therefore, consent procedures should aim to reduce bias and improve participation rates. VALiD is the first RCT to compare opt-in with opt-out parental consent for a population-based childhood vaccine safety surveillance program using data linkage. This study fills a gap in the literature in that it will not only assess the participation rate and selection bias for each consent option but, through a subsequent telephone interview of all households, will also assess consent preferences and intent compared with actual opting in and opting out behaviour, and socioeconomic factors. The findings will have relevance to all stakeholders and policy makers and will stimulate public debate about what it means to protect patients' interests.

## Abbreviations

CYWHS: Children, Youth and Women's Health Service; HREC: Human Research Ethics Committee; IRSD: Index of Relative Socio-Economic Disadvantage; NICU: Neonatal Intensive Care Unit; RCT: Randomised controlled trial; SA: South Australia; SEIFA: Socio-Economic Indexes For Areas; VALiD: Vaccine Assessment using Linked Data; WCH: Women's and Children's Hospital; UK: United Kingdom of Great Britain and Northern Ireland; US: United States of America.

## Competing interests

The authors declare that they have no competing interests.

## Authors' contributions

MSG, AJB-M and PR made substantial contributions to the conception and design of the study and obtained funding for the project. KMD was the trial coordinator and led the design and direction of the study and application for ethical approval. JGB and KMD developed and piloted the study invitation material and telephone survey and MSG, AJB-M, PR and VX contributed to the design elements. PR designed the statistical analysis for the trial. JGB developed the trial protocol, conducted interviews, collected the data and wrote the first draft of the manuscript. All authors contributed critical revisions to the manuscript.

## Appendix A: VALiD Working Group

Overall Advisory Committee

Dr Michael S Gold, Chair (University of Adelaide, SA); Prof Annette J Braunack-Mayer (University of Adelaide, SA); Prof Philip Ryan (University of Adelaide, SA); Prof John McNeil (Monash University, Vic); A/Prof Jane Freemantle (University of Melbourne, Vic); Prof Colin Thomson (University of Wollongong, Vic); A/Prof Elizabeth Roughead (University of South Australia, SA); Prof Elizabeth Elliot (The University of Sydney, NSW); Dr Lee Taylor (New South Wales Department of Health, NSW); Dr Jim Buttery (Royal Children's Hospital, Vic); A/Prof Glenda Lawrence (University of New South Wales, NSW); Dr Gary Lacey (Therapeutic Goods Administration, ACT); Dr Peter Richmond (University of Western Australia, WA); Sean Tarrant (Medicare Australia, ACT); Tony Woollacott (SA Health, SA).

Data Linkage Consent Advisory Committee

Prof Annette J Braunack-Mayer, Chair (University of Adelaide, SA); Dr Julie Leask (University of Sydney, NSW); Bernadette Richards (University of Adelaide, SA); Heather Petty (SA Health, SA); Maureen Watson (SA Health, SA); Dr Rod Givney (John Hunter Hospital, NSW); Prof Colin Thomson (University of Wollongong, Vic); Rebecca Horgan (SA Health, SA).

Study Coordinators

University of Adelaide, SA: Dr Michael S Gold, Chair; Katherine M Duszynski; Prof Annette J Braunack-Mayer; Prof Philip Ryan; Jesia G Berry; Jillian White; Vicki Xafis.
